# The Association Between Primary Open Angle Glaucoma and Clustered Components of Metabolic Syndrome

**DOI:** 10.2174/1874364101509010149

**Published:** 2015-10-06

**Authors:** Seyed Ahmad Rasoulinejad, Ali Kasiri, Mahdi Montazeri, Negin Rashidi, Maryam Montazeri, Mohammad Montazeri, Hesam Hedayati

**Affiliations:** 1 Department of Ophthalmology, Babol University of Medical Sciences, Babol, Iran; 2 Department of Ophthalmology, Jundishapur University of Medical Sciences, Ahvaz, Iran; 3 Department of Cardiology, Tehran University of Medical Sciences, Tehran, Iran; 4 Department of Internal Medicine, Tehran University of Medical Sciences, Tehran, Iran; 5 Department of Internal Medicine, Shiraz University of Medical Sciences, Shiraz, Iran; 6 Young Researchers Club, Islamic Azad University, Babol Branch, Babol, Iran

**Keywords:** Central corneal thickness, intraocular pressure, metabolic syndrome, open-angle glaucoma

## Abstract

**Purpose:**

There is conflicting evidence whether components of metabolic syndrome (MetS) increase or decrease the risk of primary open-angle glaucoma (POAG). The aim of the present study was to determine the association between metabolic syndrome and primary open-angle glaucoma.

**Methods:**

A total of 200 participants comprising 100 controls and 100 patients with POAG documented by clinical tests and examined by an experienced ophthalmologist using standard ophthalmologic equipment were included in the study. MetS was defined and based on ATP III criteria and POAG was defined by the criteria of the International Society of Geographic and Epidemiological Ophthalmology (ISGEO). The data were entered into the SPSS software and analyzed.

**Results:**

The prevalence of MetS in the glaucoma group was 53% in comparison to 38% in the control group (p=0.037). MetS was associated with an increased odds ratio for an IOP higher than 21 mmHg (OR: 1.72; 95% CI 1.03-2.79; p=0.034). The mean IOP was 24.91±4.29 mmHg in the patients without MetS, and 27.23±4.81 mmHg in those with MetS (p=0.027). The mean values of CCT were 603.64±63.16 µm in MetS patients and 579.27±72.87 µm in controls (p=0.018).

**Conclusion:**

Data showed an increased prevalence of components of metabolic syndrome in patients with glaucoma. The mechanisms underlying these associations need to be established in future studies. Our results support the recommendation that patients with metabolic syndrome undergo regular ophthalmological exams to monitor for the onset or progression of glaucoma.

## INTRODUCTION

Primary open-angle glaucoma (POAG) is a chronic and age-related disease that is the leading cause of irreversible visual disability [1]. To eliminate avoidable blindness, the International Agency for the Prevention of Blindness (IAPB) and the World Health Organization (WHO) included glaucoma in the list of priority blinding eye diseases of the VISION 2020 initiative [2]. The OAG is usually asymptomatic till advanced stages of the disease, knowing the risk factors associated with the onset or progression of OAG will help clinicians to better identify who will most benefit from screening.

Other abnormalities of glucose metabolism, including pre-diabetes and metabolic syndrome, may also be associated with glaucoma risk, but few studies have examined this issue, with conflicting results [3-5]. There are conflicting results in different studies as to whether components of metabolic syndrome, including abdominal obesity, hypertension (HTN), elevated fasting blood glucose, and hyperlipidemia, may influence the risk of OAG.

Many Iranian people have multiple components of metabolic syndrome and studies on Iranian population showed that approximately 43% of Iranian met criteria for metabolic syndrome [6]. According to the prevalence of HTN, DM, hyperlipidemia, and obesity in Iranian population, it is important for eye care providers and ophthalmologists to establish a better conception of the association between metabolic syndrome and chronic eye diseases including OAG.

The aim of the present study was to determine the association between the clustered components of the metabolic syndrome and POAG.

## MATERIALS AND METHODOLOGY

### Study Design and Participants

In this case-control study, we investigated a total of 200 Iranian subjects comprising 100 controls and 100 patients with POAG documented by clinical tests and examined by an experienced ophthalmologist, using standard ophthalmologic equipment.

The study was conducted according to the principles of the Helsinki Declaration and all participants gave their written informed consent.

POAG was defined by the criteria of the International Society of Geographic and Epidemiological Ophthalmology (ISGEO) [7]: an untreated Intraocular pressure (IOP) of 21 mmHg or more with a Goldman applanation tonometery, open anterior chamber angles on gonioscopy; glaucomatous optic disc changes (increased cup/disc ratio, thinning of the neuroretinal rim, notching) on ophthalmoscopy and visual field defects characteristic of glaucoma by standard automated perimetry with the Humphrey Visual Field Analyzer (HFA; Carl Zeiss Meditec Inc., Dublin, California). Patients included in the POAG group were shown not to have any systemic or local condition causing secondary glaucoma.

Central corneal thickness (CCT) was performed with ultrasonic pachymetry (Tomey Corporation, Nagoya, Japan).

Inclusion criteria for control subjects was IOP below 21 mm Hg, no glaucomatous changes in the optic disc, no visual field loss characteristic for glaucoma and no pseudoexfoliation material in the lens capsule or near the pupil.

The exclusion criteria were: angle closure glaucoma, high myopia (>5D), nondilating pupil, patients unable to understand a visual analog pain scale chart, history of intraocular surgery, subluxated, traumatic, and complicated cataracts.

### Anthropometric and Blood Pressure Measurements

Waist circumference (WC) was measured at the minimum circumference between the iliac crest and the rib cage over light clothing using a flexible measuring tape without any pressure to the body surface being recorded to the nearest 0.1 cm. To avoid subjective error, all measurements were taken by the same male physician for all males and the same female physician for all females.

Height (by a stadiometer using a centimeter scale) and weight (by a clinical scale) were measured in light clothing and without shoes. Body mass index (BMI) was calculated by body weight (kg)/height (m2) and BMI ≥30 kg/m^2^ defined as Obesity.

Blood pressure was measured twice after a 5min rest from the right hand in a seated position using a standard mercury manometer by certified technicians, and the mean was recorded as blood pressure.

### Laboratory Measurements

Blood samples were drawn after 10-12 hours of fasting through the antecubital vein. The samples were centrifuged within 30-45 min after collection. Fasting blood glucose (FBG), triglycerides (TG), total cholesterol (TC), low and high density lipoprotein cholesterol (LDL-C, HDL-C) were measured on fresh samples by standard kits (Pars Azmoun, Iran) by using auto-analyzer (Hitachi, Japan). FPG was measured by the enzymatic colorimetric method using glucose oxidize test. Serum TG concentrations were assayed using commercially available enzymatic reagents with glycerol phosphate oxidase. High-density lipoprotein cholesterol (HDL-C) was measured after precipitation of the apolipoprotein B-containing lipoproteins with phosphotungstic acid.

### Definition of Metabolic Syndrome

Definition of MetS in this study was based on the National Cholesterol Education Program Adult Treatment Panel III (NCEP ATP III) criteria.

ATP III criteria (the presence of any three or more of the following five symptoms) [8]:

1. Abdominal obesity: waist circumference >102 cm (men) and >88 cm (women)
2. Hypertriglyceridemia: serum triglycerides level ≥ 150 mg/dl or drug treatment for elevated TG
3. Low HDL-cholesterol:<40 mg/dl in men and<50 mg/dl in women or drug treatment for low HDL-C
4. High blood pressure: SBP ≥130 mmHg and/or DBP≥85 mmHg or drug treatment for elevated blood pressure (high BP)
5. High fasting glucose (FBS): serum glucose level ≥110 mg/dl or on treatment for diabetes

### Statistical Analysis

All data were analyzed by Statistical Package for Social Studies (SPSS) version 21 (SPSS Inc, Chicago, IL, USA). The continuous variables are reported as Mean±SD and categorical variables are presented as percentage. For the continuous variables, the data were tested for normality using the Kolmogorov-Smirnov test. Categorical variables were compared by chi-square test and the means were compared with student t-test. P-value<0.05 was considered statistically significant.

## RESULTS

A total of 200 participants (100 in the glaucoma group and 100 in the control group) were included in our study. Table **1** shows a comparison of characteristics of the study participants with and without glaucoma. Mean age was 62.44±6.71 years in the glaucoma group and 59.51±9.13 years in the control group (P=0.549). The glaucoma group comprised 34 men, while the control group included 42 (P=0.171). Lipid profile and FBS were not significantly different between two groups. There were significant differences in IOP and central corneal thickness, between the glaucoma and control groups (Table **1**).

The prevalence of metabolic syndrome in the glaucoma group was 53% in comparison to 38% in the control group. The metabolic syndrome was significantly higher in patients with glaucoma (p=0.037). MetS was associated with an increased odds ratio for an IOP higher than 21 mmHg (OR: 1.72; 95% CI 1.03-2.79; p=0.034).

The mean IOP was 24.91±4.29 mmHg in the patients without MetS, and 27.23±4.81 mmHg in those with metabolic syndrome (p=0.027). The subjects with metabolic syndrome had significantly higher IOP levels than those without metabolic syndrome. An analysis of the components of metabolic syndrome showed that the patients with elevated fasting glucose, high blood pressure and high triglycerides had significantly higher IOP levels, when compared to the subjects without these risk factors (Table **2**). The mean values of CCT were 603.64±63.16 µm in patients with metabolic syndrome and 579.27±72.87 µm in the participant without metabolic syndrome (p=0.018). Patients with high blood pressure had significantly higher mean of CCT, when compared to the subjects without this risk factor. Other components of metabolic syndrome were found not to be associated with the mean of CCT (Table **2**).

As shown in Table **3**, elevated fasting glucose, high blood pressure and elevated triglyceride were associated with glaucoma. However, there was no association between abdominal obesity and low HDL with glaucoma.

Fig. (**1**) shows that only one percent of the glaucoma patients had no metabolic syndrome component, while 12 percent had all 5 components. The odds for glaucoma seem to vary for different components of MetS, and the risk estimate increased as the number of components of MetS increased from 1 to 5. As shown in Table **4**, individuals with 4 and 5 components of the MetS had an increased OR for glaucoma: 3.76 (95% CI, 2.15-4.64) and 5.12 (95% CI, 1.58-8.39), respectively, compared with individuals with none of the components.

## DISCUSSION

The prevalence of metabolic syndrome was significantly higher in patients with glaucoma in our study. MetS was associated with an increased odds ratio for an IOP higher than 21 mmHg. Also, the subjects with metabolic syndrome had significantly higher IOP levels than those without metabolic syndrome.

Few studies have evaluated the association between metabolic syndrome or glucose metabolism biomarkers and glaucoma, with conflicting results [3-5]. In the Singapore Malay Eye Study, participants with metabolic syndrome had a lower prevalence of glaucoma [3], while the number of metabolic syndrome components was positively associated with the hazard of open-angle glaucoma in a US cohort [5].

In our study, abdominal obesity was found not to be associated with the mean IOP in glaucoma patients. There was no association between abdominal obesity and glaucoma. Some prior reports revealed that abdominal obesity and increased BMI have already been related with higher IOP [9-12]. Several studies have reviewed the direct association between obesity and OAG; some studies have reported a relationship [13, 14] while the others have not [15-17]—which is consistent with our study.

Some hypotheses deal with the relationship between obesity and OAG. First, increased intraorbital fat tissue and increased blood viscosity result in episcleral venous pressure elevation. These factors could lead to a consequent decrease in outflow facility resulting in increased IOP. This theory is supported by some studies reporting that obesity is related to increase IOP [9, 10, 12]. Second, several studies showed that hyperleptinemia, a symptom of obesity, may result in increased oxidative stress [18]. It has been shown that trabecular meshwork of patients with OAG have higher levels of oxidative damage compared to healthy subjects [19, 20]. Third theory about the relationship between increased IOP and obesity suggests that when Goldmann tonometry is done at the slitlamp on obese patients, breath holding and thorax compression may result in transitory elevations in IOP in these patients [21].

In glaucoma patients in our study, high triglycerides had significantly higher IOP levels when compared to the subjects without elevated triglyceride. It was also shown that elevated triglycerides were associated with glaucoma. However, low HDL did not show a significant relationship with IOP and glaucoma.

Tan *et al*. [3] study found a small positive association between total cholesterol and triglyceride levels and IOP. Another population-based study found an association between cholesterol and IOP, whereas a study on patients with suspected glaucoma found them to have hypertriglyceridemia [22]. An association between hyperlipidemia with elevated IOP and OAG were reported in Oh *et al*. [23] and Jaen-Diaz *et al*. [24] studies, respectively. By treating hyperlipidemia, a reduction in the risk of developing OAG has been reported in some studies [25]. A proposed mechanism indicated that statins can increase aqueous outflow facility and it is reported that in an ischemia-reperfusion rat model of the retina, statins have neuroprotective effects [26].

In many studies, HTN was shown as an independent factor affecting IOP [9, 27, 28]. Same as in our findings, some studies demonstrate a relationship between HTN and OAG [27-29]. However, this relationship was not reported in other studies [15, 30-32].

To explain the association between HTN and OAG, there are numerous theories which have been suggested. First, hypertension may cause an increase in perfusion of the ciliary artery, leading to an increase in aqueous production, resulting in higher risk of developing OAG [33]. A second hypothesis suggests that patients with HTN may have arterial damage and stiffening of the small end-vessels feeding the optic nerve. These changes might predispose patients to developing glaucomatous optic neuropathy [34]. Another theory proposes that using anti-hypertensive medications may be accompanied with episodic systemic hypotension, resulting in decreased perfusion pressure which could damage the optic nerve [35].

In the present study, an analysis of the components of metabolic syndrome showed that patients with elevated fasting glucose had significantly higher IOP levels compared to subjects with normal FBS. Also, elevated fasting glucose was associated with glaucoma.

The potential mechanisms underlying the association between glucose metabolism abnormalities and the prevalence of glaucoma in subjects without diabetes are unclear. The presence of the metabolic syndrome and elevated levels of glucose, HOMA-IR and glycosylated hemoglobin may be associated with increased levels of IOP, a key causal factor for glaucoma [16, 23]. Hyperglycemia increased fibronectin production in the bovine trabecular meshwork, which may increase the resistance to aqueous humor outflow and lead to elevated IOP [36]. Moreover, hyperglycemia could induce apoptosis in retinal neuronal cells through the hexosamine biosynthetic pathway [37]. Additionally, hyperglycemia-induced oxidative stress and advanced glycation end products may increase apoptotic death in retinal neurons [38, 39].

It has been shown in studies that DM is associated with POAG. There have been some theories that could explain the link between DM and POAG. First, there is evidence that shows that the risk of neuronal injury from stress may increase with the presence of long-standing elevated blood glucose alongside dyslipidemia [40]. Laboratory measurements have provided strong evidence for this relationship [41]. Secondly, reports indicated that the capacity to auto-regulate blood flow may decrease in diabetic eyes and so retinal blood flow will reduce in these eyes. Consequently, in response to elevated IOP, relative hypoxia occurres in diabetic eyes and levels of hypoxia-inducible factor-1 (HIF-1α) increase in retinal ganglion cells, and in the optic nerve head of human glaucomatous eyes [42].

The next theory may be related to the remodeling of the connective tissue of the optic nerve head. Studies show that DM may exacerbate connective tissue remodeling. This remodeling may decrease compliance at the trabecular meshwork resulting in an increased IOP and also in decreased compliance of the lamina cribrosa causing higher mechanical stress on the optic nerve head. Genetic factors and diabetes-related autonomic dysfunction probably contribute to this relationship [43].

## CONCLUSION

Glaucoma has a long latency period, in which glaucomatous optic nerve damage is ongoing but remains asymptomatic until later stages. The adherence to regular ophthalmological exams should be emphasized in patients with MetS, especially among those with 4 or 5 components of MetS.

Our data show an increased prevalence of components of metabolic syndrome in patients with glaucoma. Given that approximately half of the Iranian population has metabolic syndrome, the prevalence of OAG might increase in the coming years. The mechanisms underlying these associations need to be established in future studies. Our results support the recommendation that patients with metabolic syndrome should undergo regular ophthalmological exams to monitor the onset or progression of glaucoma. Further research may aid understanding of the complex interactions between metabolic abnormalities, IOP, and the risk and pathogenesis of glaucoma.

## Figures and Tables

**Fig. (1):**
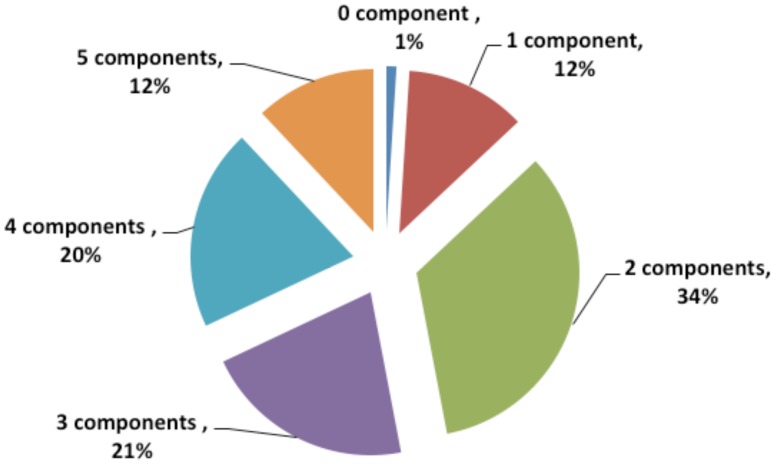
Metabolic syndrome components frequency in patients with glaucoma.

**Table 1.** Comparing baseline characteristics of the study participants with and without the glaucoma.

Characteristic	Glaucoma		*P* Value
	Yes (n=100)	No (n=100)	
Age (years)	62.44±6.71	59.51±9.13	0.549*
BMI (kg/m2)	24.31±5.76	25.42±4.96	0.631*
Gender (%)			
Males	34 (34)	42 (42)	0.171ǂ
Females	66 (66)	58 (58)	
Systolic Blood Pressure (mmHg)	137.41±19.52	120.81±19.75	<0.0001*
Diastolic Blood Pressure (mmHg)	84.39±11.54	77.51±11.28	<0.0001*
Waist Circumference (cm)	92.43±12.95	89.92±11.93	0.253*
Total Cholesterol (mg/dl)	201.54±39.89	198.34±41.73	0.641*
Triglyceride (mg/dl)	146.90±52.61	116.71±53.25	0.072*
LDL (mg/dl)	106.37±33.24	101.84±41.32	0.146*
HDL (mg/dl)	41.53±5.32	45.72±5.65	0.439*
Fasting Blood Glucose (mg/dl)	109.51±34.12	102.14±42.53	0.771*
IOP (mmHg)	26.55±4.97	17.10±3.34	<0.0001*
CCT (µm)	593.24±53.29	501.15±43.57	<0.0001*

* T-test Statistics, ǂ chi-square Statistics.

BMI, body mass index; LDL, low-density lipoprotein; HDL, high-density lipoprotein cholesterol; CCT, central corneal thickness.

**Table 2.** Comparison of the mean values IOP and CCT according to each component of metabolic syndrome participants with glaucoma.

Components	Mean IOP (±SD)	P Value	Mean CCT (±SD)	P Value
Abdominal Obesity				
Yes	26.23±4.17	0.126	591.12±56.15	0.317
No	25.34±3.76		596.34±91.77	
Elevated Fasting Glucose				
Yes	26.12±3.69	0.033	584.59±76.28	0.462
No	24.59±2.45		597.72±92.65	
High Blood Pressure				
Yes	27.24±2.62	<0.0001	601.38±74.67	0.002
No	23.83±4.47		576.23±93.79	
Low HDL				
Yes	25.37±3.23	0.234	585.53±97.28	0.538
No	26.29±2.72		598.09±64.92	
Elevated Triglyceride				
Yes	27.51±2.39	0.004	589.57±46.45	0.317
No	23.74±3.34		597.52±73.73	

**Table 3.** Associations of metabolic syndrome components with glaucoma.

Components	OR	95% Confidence Interval	P Value
Abdominal Obesity	1.12	0.72-2.01	0.151
Elevated fasting glucose	1.47	0.78-1.97	0.031
Elevated blood pressure	2.35	1.13-3.29	0.006
Low HDL	1.82	1.24-2.39	0.312
Elevated triglyceride	1.61	1.01-2.16	0.047

**Table 4.** Multivariate odds ratio of glaucoma associated with several components of MetS.

MetS Score	OR	95% Confidence Interval	P Value
0	1		1
1	1.41	0.62-3.13	0.729
2	1.97	0.95-4.01	0.162
3	2.49	1.03-4.79	0.236
4	3.76	2.15-4.64	<0.0001
5	5.12	1.58-8.39	<0.0001

## ABBREVIATIONS

BMI: = Body mass index
CCT: = Central corneal thickness
FBG: = Fasting blood glucose
HDL: = high density lipoprotein cholesterol
IAPB: = International Agency for the Prevention of Blindness
IOP: = Intraocular pressure
ISGEO: = International Society of Geographic and Epidemiological Ophthalmology
LDL: = low density lipoprotein cholesterol
NCEP ATP III: = National Cholesterol Education Program Adult Treatment Panel III
POAG: = Primary open-angle glaucoma
SPSS: = Statistical Package for Social Studies
WC: = Waist circumference
WHO: = World Health Organization
